# Extracting multiple layers of social networks through a 7-month survey using a wearable device: a case study from a farming community in Japan

**DOI:** 10.1007/s42001-022-00162-y

**Published:** 2022-03-10

**Authors:** Masashi Komori, Kosuke Takemura, Yukihisa Minoura, Atsuhiko Uchida, Rino Iida, Aya Seike, Yukiko Uchida

**Affiliations:** 1 Osaka Electro-Communication University, Neyagawa, Japan; 2 Shiga University, Hikone, Japan; 3 Bukkyo University, Kyoto, Japan; 4 Kyoto University, Kyoto, Japan

**Keywords:** Wearable device, Farming community, Social network, Non-negative matrix factorization

## Abstract

As individuals are susceptible to social influences from those to whom they are connected, structures of social networks have been an important research subject in social sciences. However, quantifying these structures in real life has been comparatively more difficult. One reason is data collection methods—how to assess elusive social contacts (e.g., unintended brief contacts in a coffee room); however, recent studies have overcome this difficulty using wearable devices. Another reason relates to the multi-layered nature of social relations—individuals are often embedded in multiple networks that are overlapping and complicatedly interwoven. A novel method to disentangle such complexity is needed. Here, we propose a new method to detect multiple latent subnetworks behind interpersonal contacts. We collected data of proximities among residents in a Japanese farming community for 7 months using wearable devices which detect other devices nearby via Bluetooth communication. We performed non-negative matrix factorization (NMF) on the proximity log sequences and extracted five latent subnetworks. One of the subnetworks represented social relations regarding farming activities, and another subnetwork captured the patterns of social contacts taking place in a community hall, which played the role of a “hub” of diverse residents within the community. We also found that the eigenvector centrality score in the farming-related network was positively associated with self-reported pro-community attitude, while the centrality score regarding the community hall was associated with increased self-reported health.

**Supplementary Information:**

The online version contains supplementary material available at 10.1007/s42001-022-00162-y.

## Introduction

**Fig. 1:**
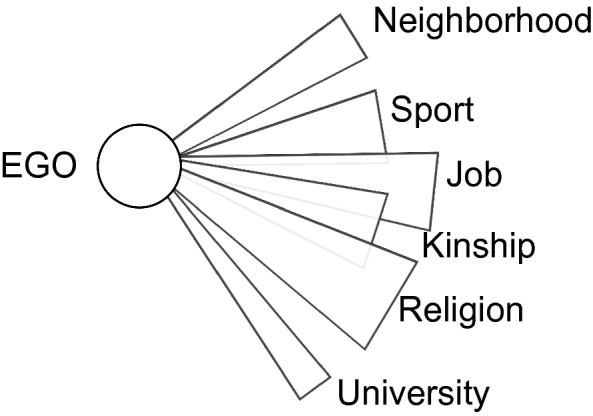
Schematic illustration of social multiplexity based on [5]. Individuals have a variety of roles in society

Human behaviors sometimes show high complexity— person who behaved nicely in one moment may express hostility in another moment, sometimes appearing to be a different person altogether [21]. The accumulated wisdom of social psychology tells us how humans are susceptible to social influences (e.g., [2, 7, 34]) and thereby behave in a context-dependent way. A person’s behavior can vary from context to context because one’s behavior is influenced by whom one is with (e.g., [27]). Furthermore, people are generally embedded in multiple networks in their daily lives. Boissevain [5] argues that people participate in several activity fields and each activity field can be regarded as a social subnetwork consisting of a set of people who potentially share a common relationship (Fig. 1). Even in a relationship between two people, it is not possible to characterize a person by a single role. Imagine a small social group of people in a given region who are mutually acquainted with each other. There exist several functional subnetworks within the group that have specific roles. Many of the group’s members participate in multiple subnetworks and may have different roles within each subnetwork. They are exposed to social influences sourced from different subnetworks and are faced with different obligations stemming from different social roles across these subnetworks. This diversity of social contexts can lead to the complexity of people’s behaviors. A relation between people who interact with each other in different positions and roles in different networks is termed multiplex or many-stranded. This kind of overlap is more common in small and isolated societies and communities [5]. To disentangle these complex behaviors, it is essential to have methods that assess the networks embedded within the social group and the roles members have within each subnetwork. In the current study, we measured interpersonal contact (proximity logs) between residents in a rural area over a long period of time using electronic devices. We aim to develop a methodology for capturing a comprehensive view of a social group by discovering the multiple subnetworks that exist in a group, based on the results of long-term proximity measurement data matrix factorization.

The problem of finding unknown subnetworks of a community has been an age-old struggle faced by many researchers. In anthropological studies, participant observations have been used to comprehensively observe the social networks of small social groups [5, 30]. However, such methods may be difficult for a study of a community of a few hundred residents. In that case, the most common survey method today is to have participants report on their own social networks in questionnaires based on recall or recognition [41]. However, the data reported by participants in these types of surveys are heavily biased and inaccurate at both the dyadic and structural levels, when compared to objective observational records, and cannot be a substitute for observational data [3, 4, 11, 28]. To conduct more reliable research, we need a method for objectively observing interpersonal contacts.

One commonly used method of objectively assessing social networks, is through Email and SNS communication logs [13, 24, 26]. However, communication often happens outside such electronic media, and face-to-face verbal and nonverbal communication also plays a strong role in maintaining social relationships. In many instances (e.g., in rural areas with many elderly people), electronic communication logs may be insufficient for revealing social networks, and a method of measuring face-to-face interpersonal interactions is needed.

One recent solution is to use wearable devices. These allow the assessment of face-to-face interactions with high temporal resolution over long periods of time [8, 12, 44]. For example, the MIT Reality Mining Project uses a device named “sociometric badges” to record interpersonal interactions between employees in a work environment over time [40]. “Sociometric badges” are devices capable of collecting multi-channel log data, including physical proximity. The strength of person-to-person proximity measured by this wearable device has been demonstrated to be linked to the subjective quality of communication [32, 42]. Using wearable devices for social network research thus allows for long-term observation, which is also useful for extracting more stable social networks than short-term observation [15].

Social network studies using wearable devices have primarily targeted interpersonal relationships in the work environment, such as within business organizations [32, 44]. However, till date, no social network survey using wearable devices has been conducted on elderly people living in rural areas. The potential value of such a wearable device is more likely to be realized in a field where research participants can move freely, rather than in a temporally and spatially confined area (such as a workplace environment). As such, this method was especially appropriate for our study, which tracked the daily interpersonal contact history of participants residing within a rural community.

Participants carried a small smartphone from morning to evening, without restrictions on time and area, so that we can collect data of diverse social interactions, not limited to specific types of interactions (e.g., business conversations).

Though data obtained from wearable devices can provide clues to reveal the structure of the community, analysis is still a dogmatic premise. Network clustering is a conventional method for revealing the structure of a social group from the records of social ties, by estimating the sub-groups to which a group’s members belong from the topography of the social connection data. Various methodologies of network clustering have been proposed [14], that ultimately serve to assign members to one or more sub-groups. However, these network clustering methods are based on cross-sectional data of social ties at a single time-point, and therefore cannot account for temporal changes in connections obtained longitudinally by wearable devices.

In this study, we adopted a different approach to extract social networks, which is based on cluster analysis, due to necessity of integrating high (temporal) resolution data logging of interpersonal interactions. These were measured over a long period of time with wearable devices. Similar to the contact-tracing applications used for COVID-19 management in some countries, the wearable devices model human interaction by communicating wirelessly with other devices within an immediate vicinity and store the information as a proximity log. The log data contain richer social information than general social network data, yielding information, for example, on who was with whom and for how long, and who was present and absent at the same time on a given occasion. To take advantage of these benefits (or using wearable devices), our study adopts an approach that resembles factor analyses. The subnetworks within a community are considered as latent common factors that cannot be directly observed, and interpersonal contact history is considered as an observation of the latent network. We also consider the social network of the entire community to be the superposition of these latent subnetworks. That is, the problem of finding unknown subnetworks of a community can be replaced by extracting common factors from interpersonal contact history.

By applying factor extraction method to the entire interpersonal contact histories of a community, it is possible to find potential common factors, or subnetworks. It is necessary to consider that the factors of these histories are non-negative, i.e., equal to 0, if no contact occurs and positive if there is contact. We, therefore, applied non-negative matrix factorization (NMF) to interpersonal contact history in this study. NMF is one of the methods to decompose matrices that have 0 or positive values [9, 25], and it has been widely applied to identify structures of various types of data, such as images, documents, genes, as well as time series data of acoustic signals. Thus, NMF is optimal for understanding the structure of social networks from interpersonal contact histories. This study attempts to discover unknown subnetworks within a community by decomposing the interpersonal contact history data into weighted rows of ties and network activity sequences using NMF. The social network created from the weighted row of ties corresponds to a set of participants who are connected to each other at the same time (we call this a subnetwork), and the network activity sequence corresponds to the activity levels of the subnetwork. One of the advantages of this method is that the latent networks can be extracted without depending on specific events or locations.

We collected proximity logs from wearable devices for 7 months. The study was conducted in a local community in Kyoto Prefecture, Japan. Data of physical proximity among community residents were analyzed by NMF to extract multiple latent subnetworks. To examine the validity of our new methodology, temporal characteristics of these latent subnetworks (e.g., pattern of temporal changes of physical proximity among community members) were examined through comparisons with qualitative data we collected through interviews with the community leader (e.g., event schedules in the community).

We also administered a questionnaire survey to the same residents to measure their subjective health and attitude toward the community (e.g., attachment to the community and trust toward other members). Several studies have suggested that pro-community attitude of residents is important for a community as it promotes the community’s crime control [35, 37] and disaster prevention [16, 31]. Through the correlations between these variables (subjective health and pro-community attitude) and the positions of residents in latent subnetworks (i.e., centrality scores), we examined (1) whether our new method can extract relevant indices in predicting important variables in the fields of social science and public health, and (2) which subnetworks are (and which subnetworks are *not*) relevant to these variables.

Our study site was a local Japanese agricultural community. Though human communities have complex overlapping multiple networks, agricultural communities are generally outstanding in this aspect. One characteristic of such communities is that people work near their homes (short or non-existent commuting times). This proximity of workplaces and residences results in co-existence of several different types of social networks in a single community, where a resident may participate in multiple networks while playing different social roles across networks. For example, one resident, Mr. A, a rice-crop farmer, would participate in a network for agricultural infrastructure maintenance with other farmers in the community. He may also participate in another network for the neighborhood watch, and perhaps an association (network) for homeowners, and a network for private social gatherings among friends. These different networks can co-exist in the same community in a complex intertwined way. The strength of Mr. A’s connection with Ms. B can be different across different networks (e.g., Mr. A and Ms. B could be close in infrastructure maintenance network, but perhaps distant in the social gathering network). As our purpose was to develop a methodology to extract latent networks from a complex accumulation of multiple interconnected networks, a local agricultural community provided an excellent context to test our idea.

Another (related) helpful characteristic of a localized agricultural community for our purpose is in its physical distance from other communities. In urbanized areas, communities and groups are generally located close to each other. In local areas, communities are relatively disconnected and traffic between them is less frequent. As a result, social relationships are relatively exclusive of individuals outside the community. Therefore, significant relationships of a resident are generally complete within their community. This completeness of networks is desirable when we try to capture influences of networks on one’s life outcomes. If an individual has many important connections with outsiders, investigating the network structures within a community does little to help us with identifying important networks for that individual.

**Fig. 2:**
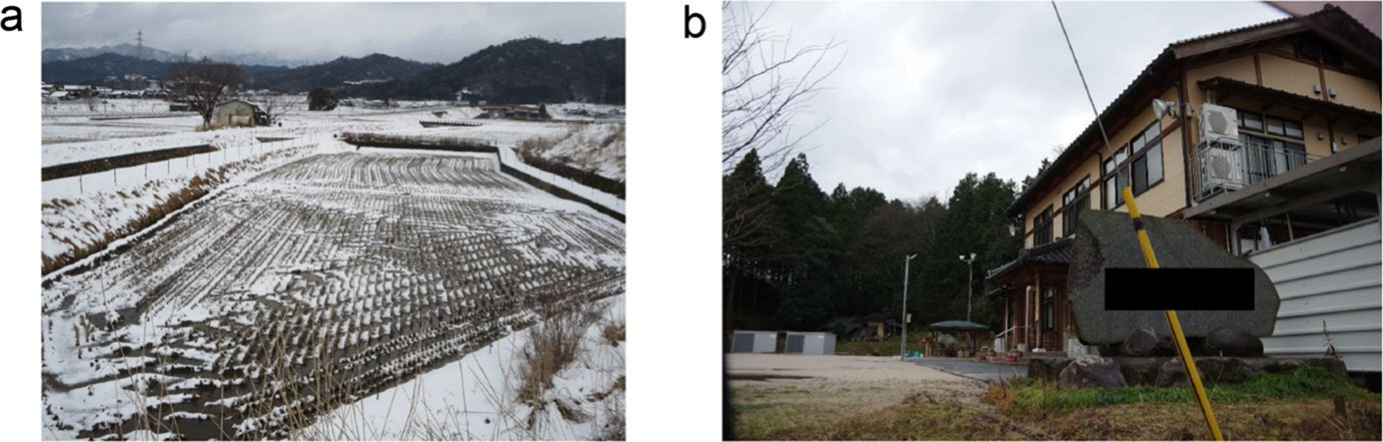
Study site, **a** landscape and **b** the community hall

## Methods

### Study site and participants

The study site was a local community (a farming village) located in a northern rural area of Kyoto Prefecture, Japan (Fig. 2). The geographical area of the community is approximately 5.0 km$^2$ [36], and the total population of the community is 840, consisting of 318 households [10], for a population density of 168 persons/km$^2$. The percentage of elderly people (aged 65 or older) is 30.5% [10]. This community is a part of a farming area, where 11.5% of the households are engaged in farming (mainly paddy cultivation) [29, 36]. To illustrate the extent to which this community is sparsely populated and small, we compared it with another community: in downtown Kyoto City (Nakagyo Ward), the population density is 14,756 persons/km$^2$ (i.e., approximately 88 times as dense as the studied community), the percentage of elderly people is 24.7%, and the percentage of individuals engaging in farming is only 0.06% [36]. Thus, when compared with this urbanized area, our studied community is less populated, more aged, and more devoted to farming. Within the accessible area for the studied community’s residents, there is only one elementary school and one junior high school, and no senior high school or university. Therefore, residents had to leave the community at least temporarily if they intended to pursue higher education.

As typical of rural Japanese communities, this community has several seasonal events. This includes a New Year’s Day celebration at a local shrine, a summer evening festival, an autumn festival, and other community-based rituals (e.g., a community gathering to make a sacred rope devoted for the local shrine at the end of a year).

Prior to the study, we conducted two pilot tests to examine the communication infrastructure (the 3G line) at the study site. We also checked the functionality of the devices and applications, and their usability for participants (especially for elders, who were often unfamiliar with using smartphones). The first pilot test (April–May 2017) was conducted with 10 participants. Following this, we upgraded the application and created a user manual to improve usability for participants. The second test (August–September 2017) was conducted with 18 participants.

After the two pilot tests (late October 2017), we started a campaign to recruit participants for the main study. Following the advice of the community leader, we distributed flyers that targeted several local groups, such as a sports team, the neighborhood watch, a social group of elderly people, and so on. The community leader helped us approach diverse range of groups so that our study would cover a broad range of people in the community. We also recruited participants at a community event (a local festival) where many locals attended. The study was explained to the residents as one that investigated people’s behavior and health. Participants would carry two small devices (wristwatch-type activity tracker and smartphone) with them and would receive the activity tracker (Go: Withings) as compensation for their participation when the data collection was completed. They were also informed that health guidance by a social worker and a doctor would be provided to participants who requested it, based on the daily step and sleep data obtained from the activity trackers and a self-report health questionnaire. Those who agreed to participate in the study received the devices, a consent form, the self-report health questionnaire (this included items on gender, age, and body size), and the user manual of the devices. The consent form and the health questionnaire were returned to the project team via post. For some participants, a DVD of a short video clip that explained how to use the devices was provided. In total, it took approximately 1 month for a sufficient number of devices to reach the community (late November 2017).

We recruited a total of 90 community residents, who received one device pair each. From this pool, we analyzed data from 58 participants, whose log data confirmed that they had been carrying the devices continuously for more than 2 months. Table 1) shows characteristics of the participants.

The study was approved by the Institutional Review Board at Kyoto University. All participants gave their informed consent. The participants provided their residential addresses to receive feedback by mail. Such identifying information was, however, accessible only to one project member who could not access the data obtained by wearable devices.

### Study period

While the study commenced on November 1, 2017, it took 1 month for a sufficient number of devices to reach the community, so for this analysis, the study period was set from December 1, 2017 to June 30, 2018.

### Procedure

#### Devices

**Fig. 3:**
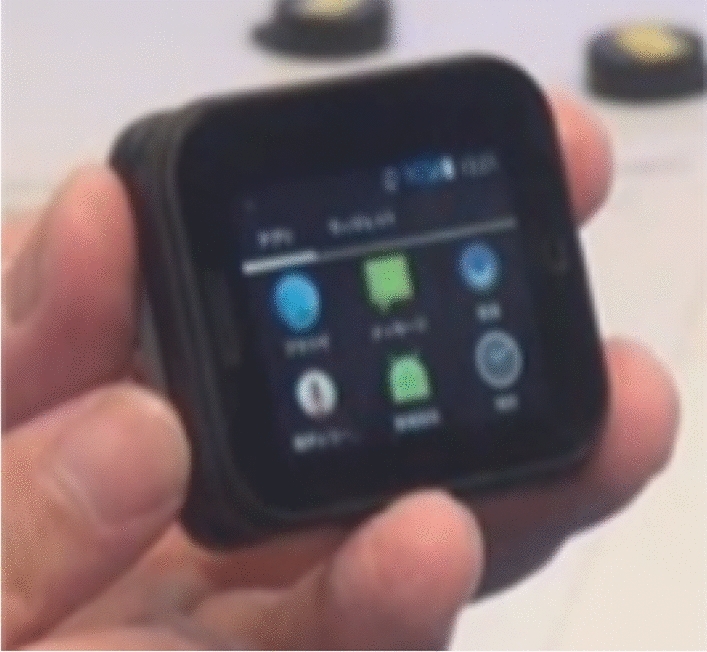
Wearable device (smartphone)

Each participant carried two devices with them: a wristwatch-type activity tracker and smartphone, of which the latter was the primary device for the current study. The smartphone (BL-01: BIGLOBE; height: 41 mm; width: 47 mm; thickness: 16 mm) was equipped with Android 4.2.2 and Bluetooth 4.0 (Class 2), and the maximum range of Bluetooth communication was 10 m (Fig. 3).

A custom-made application was installed on this terminal. This application recorded the MAC addresses of nearby Bluetooth devices and the time of detection, and the Bluetooth antenna was refreshed every 5 min. This made it possible to record the MAC addresses of other devices that were nearby (within 10 m) at 5-min intervals while the participants were out. With this application, the recorded MAC addresses and time data were uploaded to an online storage once a day via the 3G line. The activity meter data were also uploaded via the 3G network.

Participants were instructed to always carry the terminal and instructed to connect the device to a charger when they returned home. The terminal was set to restart automatically when disconnected from the charger.

To help the participants use the device properly (and to motivate them to bring the devices with them), we occasionally contacted them. In December 2017 (i.e., the first month of the data collection), we contacted participants (via telephone) whose data had not been uploaded for three consecutive days or more, and repeated explanations on how to use the device. In February 2018 (i.e., the third month of the data collection), members of the project team visited the community and gave each participant an interim report of daily step data, sleep data, and data of self-report health questionnaire along with health guidance by a social worker and a doctor. On this occasion, maintenance of the devices (e.g., battery change, application update) was carried out when necessary. We also gave the residents a brief lecture on health. Participants who could not meet the project team at this occasion received the interim report via mail. In April 2018, we provided spare devices to the community leader, in case there were some participants who needed them.

#### Self-report questionnaire on community-related attitudes

In the month of May, 2018 (the sixth month of the data collection), the participants received a paper-and-pencil questionnaire via mail (or through the community leader). The questionnaire included demographic items (e.g., occupation, educational background, and marital status) and two sets of self-report items to measure participants’ attitudes, as well as their perceptions on their community life. The first set consisted of items to assess several aspects of a participant’s positive attitude toward the community, such as community attachment, cooperative behavior toward the community, and trust toward community members (see Table 2 for the items). The items were from a series of large-scale social surveys that some of the current authors had conducted (for related studies, see [17, 18, 39] ).^1^ The second set was designed to measure participants’ openness, or attitudes toward new ideas and new people coming from outside of the community (see Table 3 for the items). These items were also from the same series of large-scale social surveys. For both sets of items, response options were on 5-point scales, with options ranging from 1 (strongly disagree) to 5 (strongly agree). In addition, the questionnaire also included an item to measure subjective health (“How would you rate your health at the present time?”) [23] and happiness (“How would you rate your current level of happiness?”) [1]. For these two items, response options were on 11-point scales, with options ranging from 0 (very bad/very unhappy) to 10 (very good/very happy). The questionnaire was completed anonymously and then returned to the project team via mail directly or through the community leader (the anonymity of the responses was maintained as the questionnaire was placed in an envelope and sealed).

**Table 1:** Sample characteristics

Variables	*n*
Gender	
Female	18
Male	39
No response	1
Occupation$^{a}$	
Full-time homemaker	6
Employed at a private business or industry	15
Self-employed	14
Employed at a public office or school	2
Farmer	9
Part-time employee	5
Retired and receiving pension payments	12
Unemployed	3
Others	2
Age	
*M* = 59.02, *Med* = 57, *SD* = 11.06, *Min* = 23, *Max* = 82	
Marital status $^{\text {a}}$	
Married	47
Unmarried	4
Divorced	1
Widowed	4
Others	1
Educational background	
Elementary school	0
Junior high school	7
High school	35
Junior college, technical college	6
University	7
Graduate school	1
Others	1
No response	1

$^{\text {a}}$ Categories are not mutually exclusive

#### Qualitative data

After the primary data collection, the project team visited the community to collect qualitative data on community activities (e.g., a festival, gatherings/meetings of the aged club, activities of a farming group) that occurred during the study period. We interviewed the community leader, the leader of the aged club, and a community hall staff.

## Results

### Factorization of proximity log data

During the survey period, the average number of times that one participant’s device detected another’s was 9.26 times/day, in which the device scanning was performed every 5 min. In this study, we assumed that when one device detected another device nearby, there was social contact between the owners of those devices. For all combinations of participants, the levels of social contacts every 30 min were scored based on the total number of device detections in the 30 min (48 epochs/day), resulting in 10,176 epochs (*I*) for the survey interval (212 days). The number of combinations (*J*) of all the participants were ${}_{58}\mathrm {C}_{2}$, as $N=58$.

**Fig. 4:**
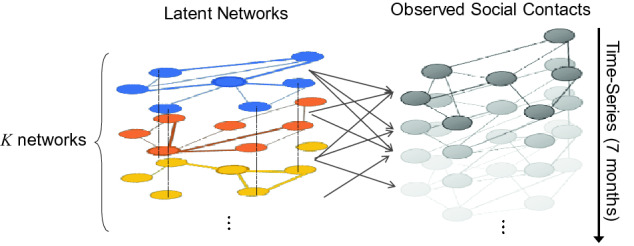
Latent subnetworks and observed network

Let ${\mathbf {Y}}$ be $I\times J$ social contact matrix, where the element $y_{ij}$ represents the number of social contacts in a combination of two participants at epoch *i*. We assume that the social contacts reflect the sum of the activities of $K(K\ll I,J)$ latent social networks with different configurations (Fig. 4). This study aims to find the latent networks in the target community by decomposing the social contact matrix ${\mathbf {Y}}$ into the product of the basis matrix ${\mathbf {H}}$ representing the time series of network activities and the coefficient matrix ${\mathbf {U}}$ corresponding to the levels of connections between two participants:

$$\begin{aligned} {\mathbf {Y}} \simeq \mathbf {HU}. \end{aligned}$$

**Fig. 5:**
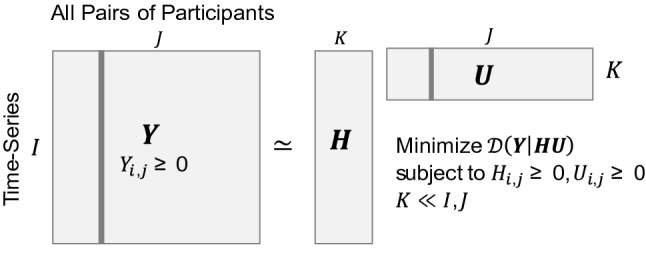
Dimensionality reduction with non-negative matrix factorization (NMF): the matrix ${\mathbf {Y}}$ is represented by the smaller matrices ${\mathbf {H}}$ and ${\mathbf {U}}$

The dimensions of the factorized matrices ${\mathbf {H}}$ and ${\mathbf {U}}$ are $I\times K$ and $K\times J$ respectively (Fig. 5). The element $h_{ik}$ of $I\times K$ basis matrix ${\mathbf {H}}$ can be regarded as the activity of the latent network *k* at epoch *i*. Thus, the matrix ${\mathbf {H}}$ shows the time series changes of the activity levels of the latent networks. The element $u_{kj}$ of $K\times J$ coefficient matrix ${\mathbf {U}}$ describes the degree of connectivity between each participant in latent network *k*.

All the elements of social contacts matrix ${\mathbf {Y}}$ are non-negative values, by requirement. Moreover, the basis matrix ${\mathbf {H}}$ and the coefficient matrix ${\mathbf {U}}$ should consist of non-negative elements, because it is natural to think that latent social networks have additive effects on social contact rather than subtractive effects. Thus, in this study, we utilized non-negative matrix factorization (NMF) to find latent networks in the community.

NMF attempts to find an approximate factorization for $\widehat{{\mathbf {Y}}}\simeq {\mathbf {Y}}$ that minimizes the distance *D* between $\widehat{{\mathbf {Y}}}$ and ${\mathbf {Y}}$. In this study, we consider NMF in which the distance *D* is measured by Euclidean distance between the matrices. The function $D_{\text {EU}}$ to be minimized is given by

1 $$\begin{aligned} D_{\text {EU}}({\mathbf {Y}},\widehat{{\mathbf {Y}}})=\parallel {\mathbf {Y}}-\mathbf {HU}\parallel ^{2}_{F}=\sum _{ij}\left( y_{ij}-\left( hu\right) _{ij}\right) ^{2}, \end{aligned}$$

where $\parallel \cdot \parallel _{F}$ denotes the Frobenius norm, and $y_{ij}\simeq (hu)_{ij}=\sum _{k=1}^{K}h_{jk}u_{jk}$ is subject to the constraints of $h_{i\alpha },u_{\alpha j}\ge 0$, where $0\le i\le I,0\le k \le K,0\le j \le J$. All computations were done within R using the package NMF [19]. The optimal number of ranks *K* was determined to be five based on the cophenetic correlation coefficient and the residual sum of squares, as well as interpretability (see below for our interpretation of each subnetwork).

**Fig. 6:**
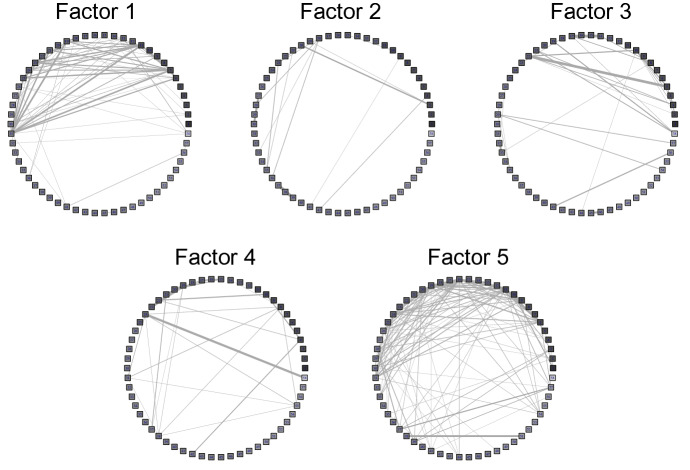
The social network corresponding to each factor. Each dot represents a participant. The darker the color, the higher the age

**Fig. 7:**
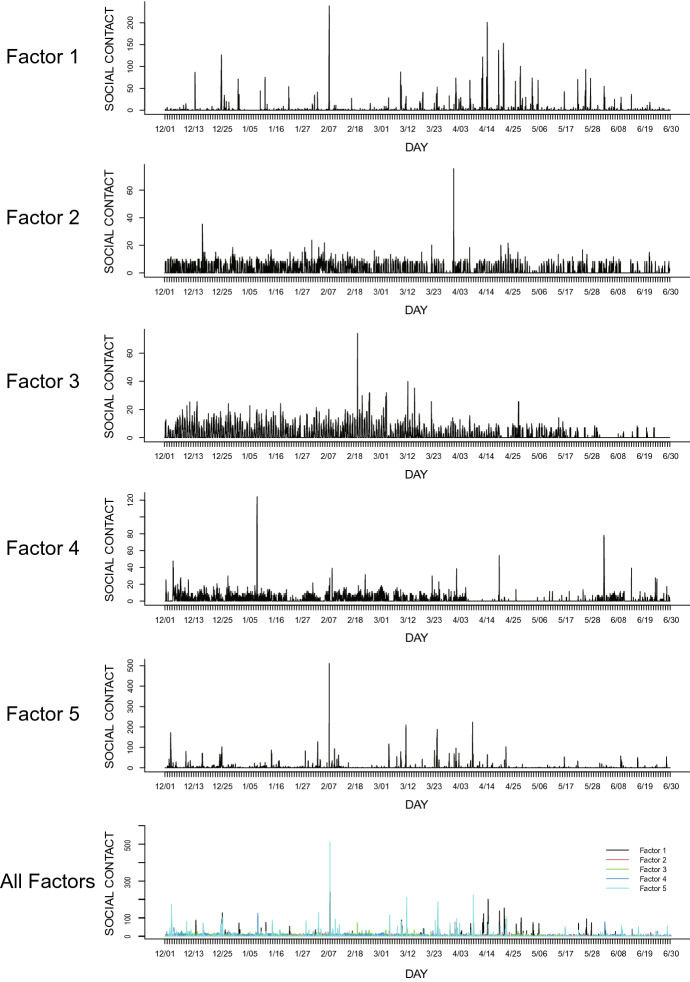
The time series of social activity levels corresponding to each factor. The bottom graph combines the graphs of all factors. The vertical axis represents the coefficients of the basis matrix ${\mathbf {H}}$. The higher the value, the more contact among the members in the subnetwork at the epoch

**Fig. 8:**
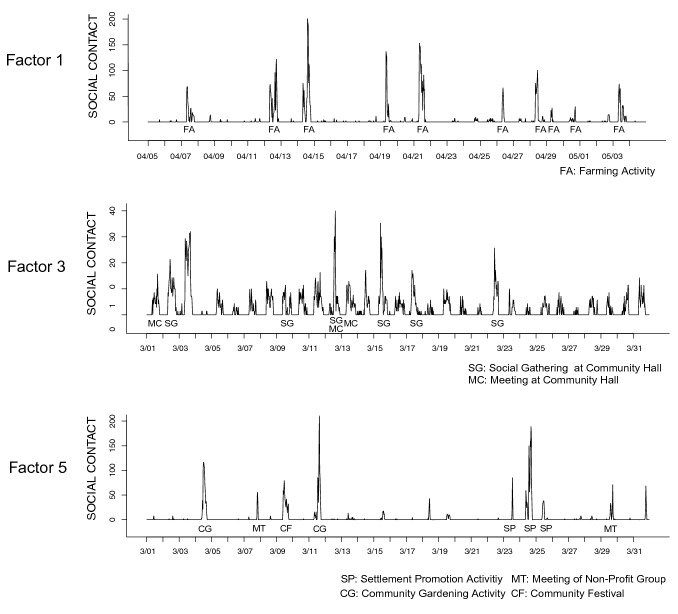
Excerpt from the base matrix ${\mathbf {H}}$, which represents the change in activity level over the month for the networks corresponding to the first, third and fifth factors. The higher the value, the more contacts among the members in the subnetwork at the epoch. Each label indicates the point when various activities took place, as revealed by the interviews. Factor 1 was associated with rice farming activities. Factor 3 was associated with activities at the community hall. Factor 5 was found to be related to various activities including community promotion activities

To elucidate the characteristics of each factor, we examine the basis matrix ${\mathbf {H}}$ and the coefficient matrix ${\mathbf {U}}$. Each column of the basis matrix ${\mathbf {H}}$ corresponds to a time series of each factor, which represents the activity level of the network associated with each factor (48 epochs/day). Meanwhile, the coefficient matrix ${\mathbf {U}}$ represents the strength of participants’ connections with each other associated with a particular factor. We reconstructed each factor and its corresponding network from the coefficient matrix ${\mathbf {U}}$ as an undirected graph (Fig. 6). Almost all the participants in our surveyed community were acquainted with each other. In evaluating the characteristics of individuals in such a small community, it is necessary to consider the importance of neighboring individuals. Therefore, we used eigenvector centrality to measure centrality [6]. Eigenvector centrality measures the importance of a node by considering the importance of its neighbors. It assigns a relative score to every node in the network based on the assumption that a connection to a high-scoring node will contribute more to that node’s score than an equivalent connection to a low-scoring node. Centrality score (eigenvector) of each participant was calculated for each subnetwork; they were log-transformed to be used in later analyses (distributions of those log-transformed centrality scores are shown by Fig. S1 in the Supplementary Material). The temporal patterns of change in the activity level of each potential subnetwork, corresponding to the basis matrix ${\mathbf {H}}$, is shown in Figs. 7 and 8. Figure 7 covers the entire study period (December 1, 2017 to June 30, 2018) for all the subnetworks. On the other hand, Fig. 8 shows time activity levels of three subnetworks that had unique patterns (discussed below) for more focused periods of time. High activity levels reflect the high proportion of members of each subnetwork participating in the activity. As shown in Fig. 7, many epochs of factors 1 and 5 have high activity levels, suggesting that these factors are associated with public events. On the other hand, factors 2 and 4 show low activity levels, indicating that they are mainly related to personal contacts.

The first factor (Factor 1) showed higher levels of activities in April (Fig. 7). According to interviews with the community leader, collaborative community activities related to rice farming, such as sowing rice seeds, transplanting rice seedlings, rice field maintenance, and weeding, occurred during the period of high activity in April. This work was mainly performed by members of a farming group in the community, and their activity schedule corresponded to the time series pattern of interpersonal contacts in Factor 1 (Fig. 8). This suggests that Factor 1 is a component related to the collaborative work of agriculture. Further, Fig. 6 shows that Factor 1 was linked to the network of relatively older participants, which is consistent with the fact that the members of the farming group were relatively old. Figure 6 also suggests that ties were not equally distributed among participants—some were densely connected while others were not connected in this subnetwork. This consistent pattern was also found from the distribution of centrality scores (Fig. S1), which shows a negatively skewed distribution. Thus, in this latent subnetwork, there was a divide among the participants in terms of the degree of connectedness with others. This is consistent with our interpretation that this factor is related to agricultural activities (e.g., transplanting rice seedlings), in that activities related to this subnetwork required some (but not all) residents’ cooperation.

The second factor (Factor 2) appeared to reflect diurnal activity, and we interpret it as representative of daily interactions with family. Figure 6 shows that the network associated with Factor 2 was composed of a small number of combinations of the participants. Similarly, Fig. S1 (histogram of log-transformed centrality score) shows that a large part of participants (approximately 40%) were located at the median (= − 6) of this distribution, suggesting that many participants were connected with some other participants to the similar degree. These characteristics of this factor are consistent with our interpretation that Factor 2 was related to interpersonal contacts among family members living together.

The third factor (Factor 3) was also associated with everyday activities (Figs. 7 and 8). A notable characteristic of this factor is that it had low-level but long-period activities, unlike the spike-like patterns shown in Factor 1. Generally, the activities started in the morning and ceased around the evening (before night), and often showed short-period reductions around noon. The level of activities was generally low, suggesting that only a few people were there at one time. Though there were some days that had higher levels of activities (e.g., March 12, 15 and 22), the interview data suggested that community activities (e.g., a social gathering of the elders’ club) occurred at the community hall on these days (Fig. 8). These patterns suggest that Factor 3 reflected interpersonal contacts among people who visited the community hall for different reasons. From the interview, the community hall functioned as a gathering point for the residents, with several different kinds of activities taking place in the hall (Fig. 2b). On weekdays, one staff member was continually stationed in the hall (from morning to evening) even without any scheduled special activities, and assisted residents who visited the hall. Therefore, we concluded that the community hall served as “hub” that connected residents directly or indirectly, for various reasons (e.g., to participate in a club activity, to meet the hall staff, to get archived documents about the community history). Given that the residents differed in the frequency of visiting the community hall, the number of chances to interact with other residents at the community hall was also different.

The fourth factor (Factor 4) was hard to interpret. Higher activity levels were observed on January 8 and June 2, 2018 (Fig. 7), but it was unclear what kind of contact these were; we could not discern any related activities from the interviews conducted. Moreover, lower activity levels were observed during the months of April and May. The centrality distribution score (Fig. S1) was not skewed, unlike Factors 1 and 5. There was no clear peak (mode) in the distribution. At this point, we are hesitant to interpret this factor. The subnetwork of Factor 4 (Fig. 6) shows that there are several small groups that are not strongly connected to each other. It suggests that there were several gatherings whose activity level decreased due to unknown factors during the same period in April and May. The activity levels of Factor 1 indicate that farming activities were more active during April and May. This agreement implies that farming activities might have suppressed the activities of the subnetwork of Factor 4.

The fifth factor (Factor 5) is associated with the network which saw involvement from many community members across a wide range of ages (Fig. 6), though age was positively associated with centrality score in this factor ($r = 0.33$; see Supplementary Material). Figure S1 suggests that despite the presence of some participants with low centrality scores, the majority of participants had similarly high scores for centrality. High activity levels (Figs. 7 and 8) coincided with community festivals, meetings of local non-profit groups, bazaars, community gardening activities, and settlement promotion activities (inferred from interviews with the community leader). This suggested that Factor 5 was a component closely connected to community promotion activities, which was organized by the community promotion committee and attended by many residents.

Another major activity was seen on February 7 in all the five factors (Fig. 7). This was the day the project team visited the community to conduct interviews with the participants and provided healthcare information.

Table 4 shows descriptive statistics of the centrality scores of the latent networks as well as the self-report scales. Table 5 shows the correlations between them. All the centrality scores of latent networks were positively correlated with each other. Among them, the strongest correlation was found between Factor 1 (activities of farming group) and Factor 5 (community promotion activities), suggesting that residents who were located at the center of the network for farming activities were also located at the center of the network for community promotion activities.

### Reliability of self-report scales

The internal consistency of items for measuring positive attitude toward the community (pro-community attitude) is shown in Table 2. We used a principal component analysis (PCA) to assess the internal consistency of the nine items for measuring pro-community attitude and the five items for openness, separately. For pro-community attitude, two items had low factor loadings (see Table 2) and thus were excluded. The remaining seven items showed sufficiently high Cronbach’s coefficient alpha (0.88) and McDonald’s coefficient omega (0.91). “Pro-community attitude” was computed by averaging seven items. See footnote^2^ for validation checks of this composite measure. The item “I participate in community activities (e.g., meeting and events),” which was not included in the pro-community attitude score, was also used in the analyses below as a separate item measuring “participation in community activities.”

For openness, one item with low factor loading was excluded (Table 3) and the remaining four items showed acceptable Cronbach’s coefficient alpha (0.67) and McDonald’s coefficient omega (0.80). These were averaged to provide a measure of “openness”. See footnote^3^ for validation checks of this composite measure.

**Table 2:** Internal consistency of items for measuring pro-community attitude

	PCA loading	
	9 items	7 items
I feel attached to my community.	0.85	0.85
The community should maintain their local traditions inherited from the past	0.81	0.80
I trust the people who live in my community.	0.81	0.84
The people in my community basically act honestly.	0.76	0.76
I try to always follow the established rules of the community.	0.69	0.69
If people in the community need help, I help them.	0.69	0.72
I treat my neighbors to food, taking them out to eat and/or inviting them over for a lunch/tea/dinner$^{\text {a}}$	0.68	0.68
I participate in community activities (e.g., meeting and events)	0.56	–
I think that I should not refuse a request made by someone in the community who has helped me or done something nice for me	0.39	–
Cronbach’s $\alpha$	0.87	0.88
McDonald’s $\omega$	0.90	0.91

$^{\text {a}}$ This is a popular and prosocial behavior in Japanese local community

**Table 3:** Internal consistency of items for measuring openness

	PCA loading	
	5 items	4 items
We should incorporate different values and ways of thinking from outside our own community	0.83	0.84
We should create a new culture and not be bound by tradition	0.66	0.67
I would be happy if a person from another country settled in my community	0.75	0.73
I would be happy if a person from outside of my community settled in this community	0.59	0.58
If more people moved into this community from other places, some problems would increase	− 0.09	–
Cronbach’s $\alpha$	0.56	0.67
McDonald’s $\omega$	0.72	0.80

**Table 4:** Descriptive statistics

	*N*	Mean	Median	SD	Min	Max
Factor 1 (activities of farming group)	58	− 2.99	− 2.54	2.21	− 8.73	0.00
Factor 2 (family contacts)	58	− 5.52	− 5.98	2.20	− 12.60	0.00
Factor 3 (contacts at the community hall)	58	− 5.34	− 5.59	2.26	− 13.17	0.00
Factor 4	58	− 4.85	− 5.13	2.25	− 11.40	0.00
Factor 5 (community promotion activities)	58	− 2.08	− 1.54	1.87	− 8.42	0.00
Pro-community attitude	60	3.62	3.64	0.68	1.71	5.00
Participation in community activities	62	3.94	4.00	0.74	2.00	5.00
Openness	59	3.61	3.50	0.64	2.00	5.00
Happiness	59	7.02	7.00	1.62	3.00	10.00
Subjective health	61	6.72	7.00	1.60	3.00	9.00

**Table 5:** Correlations between the log-transformed eigenvector centrality scores and self-report scales

	(1)	(2)	(3)	(4)	(5)	(6)	(7)	(8)	(9)
(1) Factor 1 (activities of farming group)	–								
(2) Factor 2 (family contacts)	0.32$^{*}$	–							
(3) Factor 3 (contacts at the community hall)	0.38$$^{**}$$	0.39$$^{**}$$	–						
(4) Factor 4	0.44$$^{**}$$	0.39$$^{**}$$	0.46$$^{**}$$	–					
(5) Factor 5 (community promotion activities)	0.63$$^{**}$$	0.41$$^{**}$$	0.36$$^{**}$$	0.49$$^{**}$$	–				
(6) Pro-community attitude	0.27$^{*}$	0.15	0.17	0.28$^{*}$	0.16	–			
(7) Participation in community activities	0.12	− 0.02	0.11	− 0.03	0.05	0.46$$^{**}$$	–		
(8) Openness	0.07	0.11	0.22	0.22	0.08	0.06	0.23$\dag$	–	
(9) Happiness	0.22	0.05	0.11	0.07	0.22	0.23 $\dag$	0.10	0.00	–
(10) Subjective health	0.15	0.22	0.39$$^{**}$$	0.00	0.22	0.21	0.24$$^\dag$$	0.26$$^{*}$$	0.48$$^{**}$$

$$\dag$$ $$p<0.10$$

$$*$$ $$p<0.05$$

$$**$$ $$p<0.01$$

### Correlations between centrality scores in latent networks and self-report scales

Among the self-report scales, pro-community attitude was positively correlated with participation in community activities (Table 5). Pro-community attitude was also positively correlated with happiness but only marginally. Participation in community activities also had weak (marginally significant) positive correlation with openness and subjective health. Openness and subjective health were positively correlated with each other. Finally, subjective health was positively correlated with happiness.

Our primary focus was toward understanding the relationship between pro-community attitude and centrality scores of latent networks. Centrality in the Factor 1 network (activities of farming group) was positively associated with pro-community attitude. Factor 2, which presumably reflected network among family members, did not have any significant correlation with the self-report scales. Centrality in the Factor 3 network (contacts at the community hall) was positively associated with subjective health. Centrality in the Factor 4 network, an unknown network, was also positively correlated with pro-community attitude. Centrality in the Factor 5 network (community promotion activities) did not have any correlation with the self-report scales. Participation in community activities (self-report) did not correlate with any centrality scores of latent networks. We will discuss this in “Discussion”.

## Discussion

With the development of IoT technology, the process of obtaining detailed data on people’s spatial proximity over time has become increasingly accessible. Social network surveys using such IoT technologies have higher ecological validity than surveys using paper questions or interviews, because they capture the real day-to-day behavior displayed by survey participants and is free of response and recall biases. However, in communities with complex interpersonal relationships, where multiple social networks overlap, it remains a challenge to discover interpretable social networks from large amounts of long-term, digitally recorded data. This may be partially due to the insufficient use of temporal information in social network studies using wearable devices. In this study, we aimed to extract latent social subnetworks in a local community by factorizing the time series log data matrix of the spatio-temporal proximity using NMF, and to evaluate the interpretability of the extracted latent network. We conducted a 7-month study using wearable devices in a farming community in Japan. This dataset provides rich information on changes of interpersonal contacts—not only micro-scale changes (i.e., changes within 30 min), but also macro-scale (seasonal) changes. Seasonal differences are especially important given that several activities in farming communities are season dependent. In addition, our dataset itself is an important contribution, as it consists of a wide range of age groups in a (relatively) isolated population, and are generally harder to access for researchers beyond university students or crowdsourced workers who are commonly used in this discipline.

We extracted five latent subnetworks from proximity logs. The proximity logs were decomposed into a basis matrix (corresponding to the temporal activity patterns of each subnetwork) and a weight matrix (corresponding to each tie between the members). We found that the extracted subnetworks showed reasonable and interpretable temporal patterns, suggesting the validity of our method. For example, Factor 1 showed a time series pattern of social contacts that tracked the activities of the farming group. Factor 1 showed high levels of activities around early April, where farming activities are generally busy. At a more fine-grained scale, high levels of activities were observed exactly on days when the farming group’s activities (e.g., transplanting rice seedlings) were carried out. The other subnetworks had unique characteristics, and some of them showed interpretable patterns (e.g., Factor 3 seemed associated with contacts among a wide range of residents at the community hall).

We also found that the extracted subnetworks provided useful information in predicting important variables in the fields of social science (pro-community attitude) and public health (self-rated health). We measured these variables by the self-report scales and examined which subnetworks are (and which ones are not) relevant to these variables. As a result, we found that the centrality score of Factor 1 was positively associated with scores of pro-community attitude. That is, individuals at the center of the farming-related social network were more likely to be involved in reciprocal/cooperative relationships in the community than other individuals. This finding is consistent with previous studies showing that farming is connected to several collective activities in communities such as collective works to maintain shared facilities (e.g., [39]).

Interestingly, the farming-related network was not the only one sustaining reciprocal/cooperative relationships in the community. The centrality score of Factor 4 was also associated with pro-community attitude, suggesting that this unknown subnetwork is essential for cooperative relationships in the community. Yet, the time series pattern of this subnetwork did not correspond to the dates of community activity that we learned from the interview with the community leader and others. One interpretation could be that Factor 4 perhaps reflects more casual gatherings of informal groups and random encounters, which may play important roles to maintain cooperative relationships [33]. From another perspective, it may be that a person’s high frequency of random contacts (rather than appointed ones) with others reflects how deeply their daily activities are intertwined with those of other residents (and thereby tended to show higher pro-community attitude). Either way, this unexpected association suggests that our method helps reveal social networks that are hidden yet play important roles in communities.

However, not all the subnetworks were related to pro-community attitude. For example, the centrality score of Factor 2 (family contacts) was not correlated with pro-community attitude. In addition, unexpectedly, the self-report measure of participation in community activities did not correlate with any of the centrality scores (Table 5) including Factor 1 (activities of the farming group). If the farming group plays central roles for reciprocal/cooperative relationships in the community (as seen in the correlations between pro-community attitude and Factor 1 centrality score), those with high centrality in Factor 1 should show the greater tendency to participate in community activities than others. One possibility has to do with a ceiling effect for the item of participation in community activities. The item might assess light commitment to the community (e.g., dropping by a community festival) rather than more heavy commitment (e.g., involving in the festival as a staff). In fact, the median of this item was relatively high (4.00 in the five-point scale ranging from 1 to 5) compared to pro-community attitude (median = 3.64). If that was the case, even though members of the farming group committed more deeply to participation in community activities than other residents, the current self-report item for participation in community activities failed to capture such a difference. This implies that we need an item asking how deeply one engages (rather than asking about participation) in community activities if researchers are interested in individual differences in the involvement in agricultural communities, where participation in community activities is generally high compared to other types of communities (e.g., [39]).

Self-rated health was positively correlated with Factor 3. Factor 3 presumably reflected a wide range of interpersonal contacts occurring at the community hall. The community hall played the role of a “hub” in this community. Different groups in the community visited the hall either regularly, or on an ad-hoc basis. For example, the elderly group had regular meetings at the hall. A group of women regularly gathered at the hall to conduct exercise sessions, and several people gathered there for occasional drinking sessions. The hall might be a place to connect different groups and diverse residents from the community. Then, Factor 3, which traced interpersonal contacts at the community hall, might be a network covering a wider range of social relationships in the community than the other subnetworks. If so, it is understandable that a person who cannot engage in even such a network may have a health issue that prevents them from commuting to the hall. Taken together, the findings suggest that our method can extract a social network that helps us identify individuals who may be of poor health.

### Implications

First, the current study showed that spatio-temporal proximity data over a long time recorded by wearable devices is useful to detect meaningful structure of social networks. This is important given that self-report methods of interpersonal contacts have a non-negligible limitation that it cannot capture unnoticed/unmemorable interpersonal contacts [3, 4, 28]. Such contacts can still be a significant source of social influence on human minds [20] and thus need to be quantified. Using wearable devices for a long period can be a solution for this methodological issue. In fact, the Factor 4 subnetwork, which was seemingly representative of hidden social networks, was linked to pro-community attitude.

Second, the current study provides a novel method to extract complex and multi-layered social network structures. By factorizing proximity data, we were able to extract multiple (and mutually overlapping) latent subnetworks. This method is especially useful when researchers try to unravel complex interwoven ties among community members. People are often embedded in multiple social networks simultaneously (e.g., a researcher may be involved in multiple collaborative research projects, while teaching multiple courses, on top of working as a committee member for the university administration), and these networks are somewhat overlapping (e.g., one of their colleagues in the committee is also a member of the research project). By capturing such different types of networks simultaneously, we can examine what kind of networks (e.g., casual network, formal network) can be a channel for the transmission of various types of social influences (e.g., [7]).

Third, the current study provides a new perspective on social networks. Traditionally, studies have developed methods to classify individuals into clusters (e.g., [14]) and assess networks among them. In the current study, we proposed a new method to classify ties (or proximity) into latent subnetworks. This approach is based on a perspective to view proximities as observations that reflect latent structures behind of them. By viewing interpersonal contacts in this way, the multi-layered nature of social relationships in the real world can be targeted in empirical investigations. Under this view, it is only natural that individuals are embedded in multiple (and possibly overlapping) subnetworks and are sometimes forced to play different social roles across various contexts. As different social roles sometimes place conflicting obligations on an individual, the multi-layered nature of social networks is an important research theme regarding social stress that people face in their daily lives.

### Limitations and future directions

First, our sample size was not large, and our efforts to recruit participants did not necessarily cover the entire community. We largely relied on the community leader’s direct and indirect connections to recruit participants. This limitation comes from our decision to conduct the study in a farming community, in which social networks likely overlap and are interwoven complexly. As smartphones were not so common in such a community at the time of the survey, we needed to distribute the devices and asked the participants to bring them every day. Given that the devices were not necessarily familiar to the participants, this was not an easy request to accept. Therefore, we had to rely on the community leader, as the most influential person in the community, to recruit participants. Future studies with a larger sample and wider range of participants in a target community are needed. Alternatively, given the difficulty to collect network data over a long period of time, each study can be replicated at a smaller level with a relatively smaller sample size. By accumulating data from such studies, researchers can perform a meta-analysis, which would help overcome any problem caused by a small sample size and help conduct more fine-grained analyses as well (e.g., examination of possible moderating effects by gender). If accumulated data come from different types of communities, meta-analysis would also enable the examination of the generalizability of the findings across different contexts. The current study can be a part of such accumulation.

Second, on a related note, we could not ensure that participants brought the devices with them every time they left the house. When not physically with their devices, any encounters with other participants would be missed. One solution could have been to use participants’ own smartphones, but with the low rates of smartphone ownership, this was another limitation of our decision to conduct the study in a farming community. For this study, a farming community provided an ideal circumstance for our research question (that is, complexly overlapping subnetworks), but future studies in more urbanized settings would be useful to examine the applicability of our method to different settings.

Third, like most factor analyses, deciding on the number of factors (subnetworks) was a challenge. In our case, the number of factors was determined not only by referring to the cophenetic coefficients and the residual sum of squares, but also by considering the interpretability of qualitative data obtained through the interview. Yet, it is still difficult to narrow down interpretations (e.g., we cannot be sure if “contacts at the community hall” really occurred at the community hall). In future research, the utilization of GPS location data may provide useful information for determining the appropriate number of factors. This study only measures physical proximity data and does not measure communication via PC or smartphone. Therefore, we were unable to examine whether or not there exists a subnetwork that is based on electronic communication, though the existence of such a subnetwork is possible. In future research, it should be important to examine the differences and interactions between social networks based on electronic communication and networks based on face-to-face communication.

Finally, to measure participants’ health, we relied on self-reported questionnaires. As there would be an issue of reference group effect [22], the results should be interpreted with caution (e.g., elderly people might rate their health in comparison with other elderly people, not younger people). Future research should consider using biological markers to assess participants’ health.

### Conclusion

Human society often comprises several, multi-layered, complex social networks. To understand interpersonal behavior, we must first disentangle such complexity and extract interpretable subnetworks. To this end, the current study proposed a new method using NMF. This method successfully extracted five subnetworks from a 7-month survey of a farming community of Japan, that used wearable devices to track instances of social interaction. The extracted subnetworks helped predict individual differences within the community along the levels of pro-community attitude and health. The study contributes to the literature by adding a new method and a new perspective to comprehend face-to-face social interactions and structures of latent social networks that explain these interactions.

## Supplementary Information

Below is the link to the electronic supplementary material.

Supplementary file1 (PDF 201 KB)

## Data Availability

The datasets generated during the current study are not publicly available owing to privacy issues. However, they are available from the corresponding author upon reasonable request.

## Footnotes

1 The series of large-scale social surveys were conducted in more than 500 communities mainly located in the western part of Japan. All surveys used paper-and-pencil questionnaires, and most of them were delivered via mail to residents in the target communities. The communities were diverse, including urban, farming, and fishing communities, scattered across different geographical areas of Japan. The surveys were conducted as part of a multiple-purpose project. Therefore, each survey needed to measure many concepts, and hence could not have many items for each one. As such, the project team developed items to measure several different aspects of participants’ community life by a small number of items. The items we used in the current study came from the surveys of this project. Some of them were created by modifying items from standardized scales (e.g., the generalized trust scale of Yamagishi and Yamagishi [43]). We chose items to assess several aspects of a participant’s positive attitude toward the community and examined their combinations (as described in “Results”). We took the same approach for the openness measure as well.

2 Two surveys from the aforementioned large-scale project (see footnote 1) can provide data to examine the validity of this composite measure of our pro-community attitude. One survey (“survey 1”) collected data from 6409 individuals from 533 communities and used the same six items for pro-community attitude as the current study (the item “I try to always follow the established rules of the community” was not used in this survey). The other survey (“survey 2”) collected data from 1066 individuals from 91 communities and used the same seven items for pro-community attitude as the current study. These two datasets showed that the internal reliability of this composite measure was high ($\alpha s = 0.77$ and 0.81 in surveys 1 and 2, respectively). In addition, these two datasets showed that the composite measure of pro-community attitude had reasonable correlations with other variables. For example, the pro-community attitude score was positively correlated with how long a participant had lived in the community (*r* = 0.25, *p* < 0.001 in both surveys 1 and 2). Participation in collective activities in the community (e.g., maintenance work on public facilities, disaster-prevention group activities; see [39]) was also positively correlated with the pro-community attitude score ($rs = 0.30$ and $0.31, ps < 0.001$ in surveys 1 and 2, respectively). These findings are supportive of our approach combining the seven items to create a measure of pro-community attitude.

3 The same two surveys (see footnote 2) can also provide data to examine the validity of this composite measure of openness. Survey 1 used the same four items for openness as the current study. Survey 2 used the same three items for openness as the current study (the item “I would be happy if a person from another country settled in my community” was not used in survey 2). These two datasets showed that the internal reliability of this composite measure was not necessarily high as in the current study ($\alpha s = 0.59$ and 0.58 in surveys 1 and 2, respectively). Yet, the composite measure of openness had reasonable correlations in these datasets. The surveys had two items from a scale of innovation-promotive behavior [38]. The two items were originally designed to measure a behavioral tendency to make proposals for a company (in surveys 1 and 2, the items were modified to fit in the context of local communities). The composite measure of openness was positively correlated with this active behavioral tendency ($rs = 0.22$ and $0.38, ps < 0.001$ in surveys 1 and 2, respectively).
