# Supplementary material for: Extracting multiple layers of social networks through a 7-month survey using a wearable device: a case study from a farming community in Japan
Source: J Comput Soc Sci. 2022 Mar 10;5(1):1069–94. doi: 10.1007/s42001-022-00162-y (PMC8908302; doi:10.1007/s42001-022-00162-y)
Supplement: Supplementary file 1 — Supplementary file1 (PDF 201 KB) [file 42001_2022_162_MOESM1_ESM.pdf]

Supplementary Materials for

Extracting multiple layers of social networks through a seven-month survey using a wearable device: A case study from a farming community in Japan

This file includes:
- S1. Histograms of centrality scores of latent subnetworks
- S2. Age and centrality scores of latent subnetworks
- S3. Gender and centrality scores of latent subnetworks

S1. Histograms of centrality scores of latent subnetworks

Figure S1 shows the histograms of log-transformed centrality scores of the five latent subnetworks. Note that a log-transformed score has negative values (i.e., < 0) when the corresponding raw score is smaller than 1, and the upper limit in the raw score of our measure (eigenvector centrality) is 1.

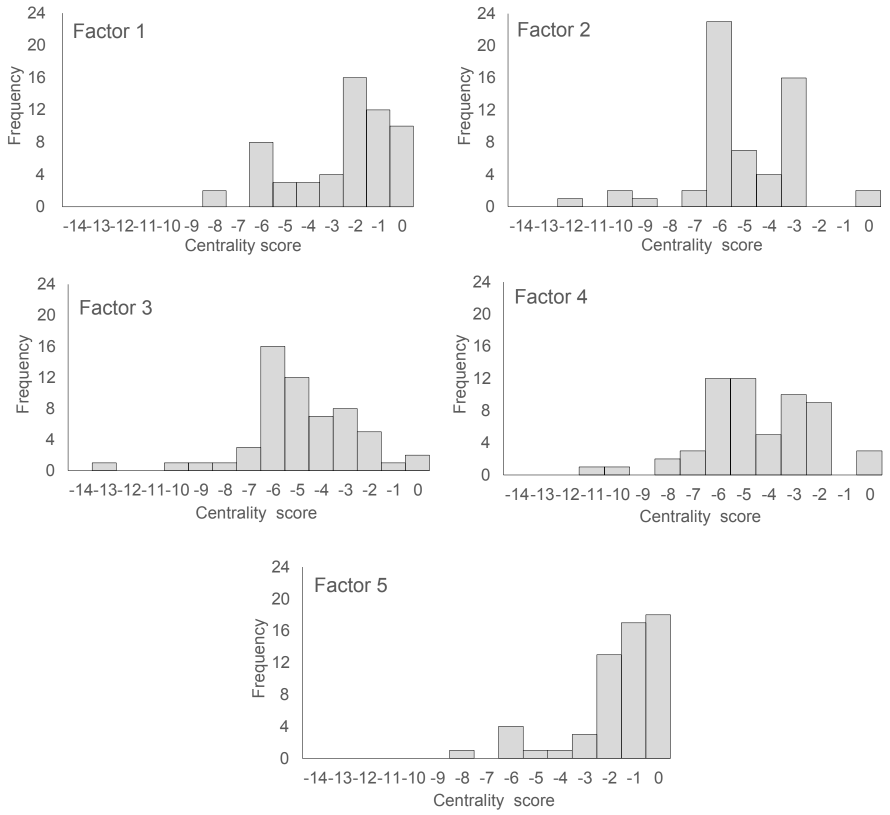

Fig. S1 Histograms of the log-transformed centrality scores of the latent subnetworks

S2. Age and centrality scores of latent subnetworks

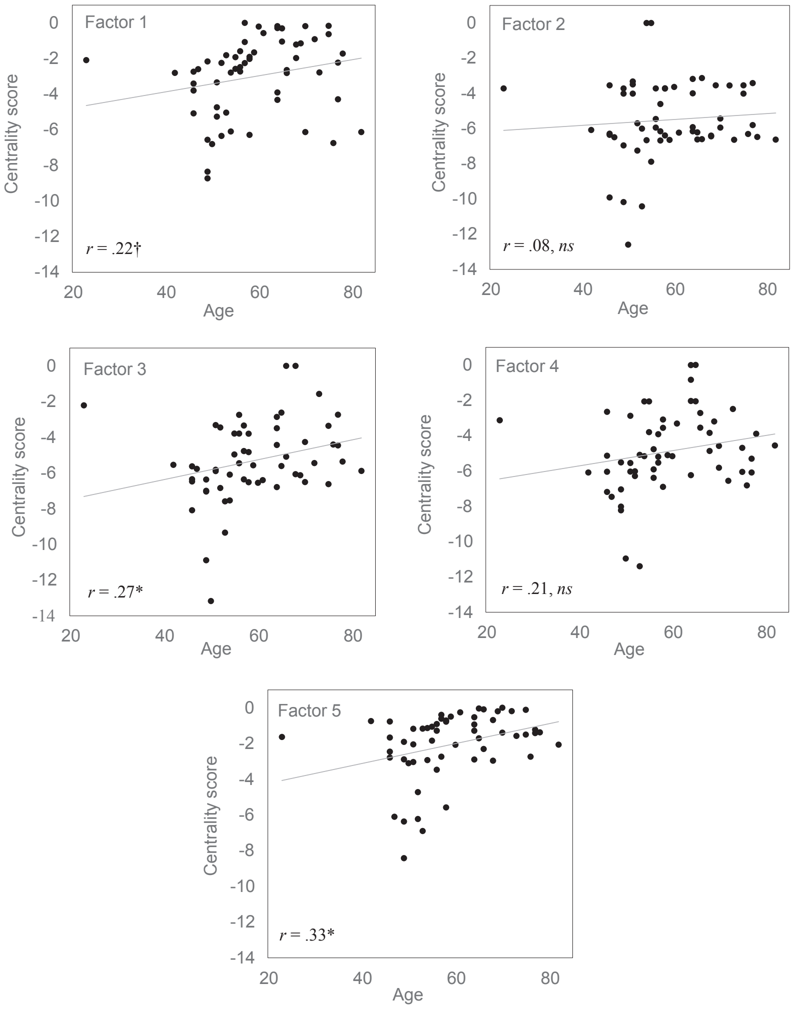

Fig. S2 Plots of the log-transformed centrality scores of the latent subnetworks as a function of age. The gray lines are fit lines. $\dagger p < .10, *p < .05, **p < .01$.

S3. Gender and centrality scores of latent subnetworks

Figure S3 shows the violin plots of log-transformed centrality scores of the five latent subnetworks as a function of gender. Table S3 shows their means and standard deviations. A significant gender difference was found only for Factor 1, where males tended to have a higher centrality score than females ($t(55) = 2.33, p = .023$).

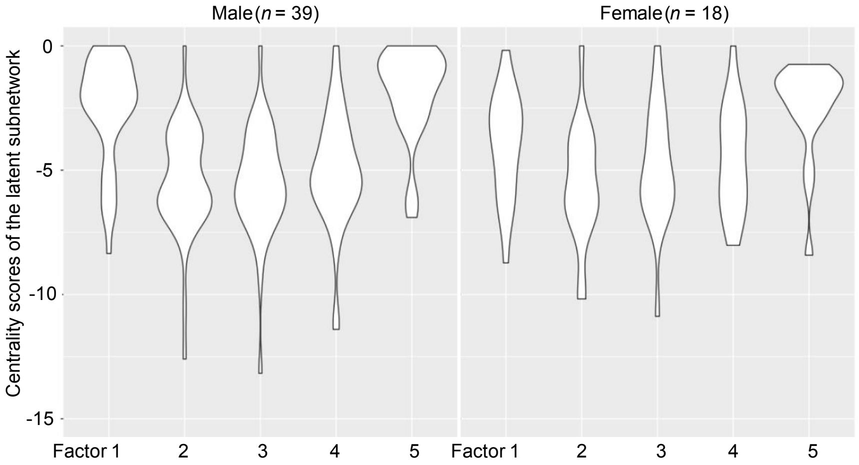

Fig. S3 Violin plots of the log-transformed centrality scores of the latent subnetworks as a function of gender.

Table S3 Log-transformed centrality scores of the latent subnetworks by gender

	Male		Female	
	M	(SD)	M	(SD)
Factor 1	-2.57	2.14	-3.99	2.13
Factor 2	-5.47	2.16	-5.56	2.40
Factor 3	-5.51	2.19	-4.88	2.45
Factor 4	-5.05	2.29	-4.56	2.14
Factor 5	-1.86	1.85	-2.51	1.95
